# A Three-Dimensional Laser Scanning-Based Method for Dimensional Inspection of Large-Scale High-Speed Railway Precast Box Girders

**DOI:** 10.3390/s26123657

**Published:** 2026-06-08

**Authors:** Zhiguo Zhang, Shihao Dou, Shaopeng Zhang, Kang Chen

**Affiliations:** 1 School of Civil Engineering, Shijiazhuang Tiedao University, Shijiazhuang 050043, China; zhangzhg@stdu.edu.cn (Z.Z.); 1202401151@student.stdu.edu.cn (K.C.); 2 China Railway Design Corporation, Tianjin 300142, China; zhangshaopeng@crdc.com

**Keywords:** high-speed railway prefabricated box girder, exterior dimensional inspection, 3D laser scanning, point cloud data

## Abstract

We present a 3D laser-scanning method for the fast, accurate dimensional inspection of large high-speed-rail precast box girders. The pipeline uses low-pass filtering plus sequential registration to suppress noise, and voxel filtering with curvature-aware enhancement to reduce point cloud size by 3–5× while preserving key geometry. Reconstruction employs K-nearest-neighbors and PCA to detect boundaries and curvature jumps, B-spline fitting with moving least squares for surface completion, and CSS corner detection to extract key dimensions at millimeter precision. Field tests report absolute errors ≤ 2.0 mm versus manual measurement, validating the method for automated, digital acceptance.

## 1. Introduction

Prefabricated, simply supported box girders, due to their advantages of high stiffness, good integrity, and short construction periods [1,2], are the preferred structural form for bridge construction in China’s high-speed rail network. They currently account for over 95% of the commonly used simply supported beams in prefabricated erection [3]. As the standards for passenger-dedicated rail lines continue to evolve, higher demands are placed on construction quality and the accuracy of exterior dimensions. For example, excessive deviation in prefabricated girder length can affect the erection quality and lead to issues with expansion joints; and insufficient concrete wall thickness reduces the protective layer depth, making reinforcement more susceptible to corrosion and spalling, which in turn compromises the durability and safety of the structure and increases operation and maintenance costs. Therefore, precise control over the overall and cross-sectional dimensions of prefabricated girders is critical.

Current dimensional inspection of prefabricated high-speed-railway girders predominantly relies on traditional contact measurement tools such as steel tape measures, calipers, and leveling instruments. These manual inspection methods exhibit three notable shortcomings [4]: first, single-point contact measurements are inefficient and cannot meet the time-efficiency requirements of industrialized, batch production; second, the intrinsic accuracy limits of mechanical gauges, compounded by operator-dependent variability, undermine data reliability; third, conventional approaches capture only local geometric parameters and are therefore incapable of producing comprehensive, full-feature digital models of the girder. In this context, three-dimensional (3D) laser scanning, owing to its non-contact, full-surface measurement capability and high-density point cloud acquisition, offers an innovative solution for the high-precision quantitative analysis of engineering geometry [5,6,7]. Field applications have demonstrated successful deployments of this technology in deformation monitoring of high-speed-railway tunnels and 3D modeling of large underground spaces, delivering millimeter-level accuracy and comprehensive feature coverage; consequently, 3D laser scanning has become a key enabler of the surveying profession’s transition from two-dimensional to three-dimensional digital workflows [8,9], and provides a viable intelligent method for the dimensional inspection of prefabricated high-speed-railway girders.

With the rapid development of three-dimensional (3D) laser scanning, research on its engineering applications has been primarily concentrated on geometric feature analysis [10,11]. The technological framework centers on reverse modeling, whereby high-precision point cloud data are used to reconstruct structural geometry and thus enable millimeter-level resolution of engineering parameters [12,13]. In the field of dimensional inspection for the prefabricated concrete box girders used in high-speed rail, studies employing laser point cloud scanning for outer-contour recognition and appearance-dimension measurement hold considerable engineering value [14]. Existing work indicates that point cloud-based reverse modeling and feature-extraction techniques offer innovative alternatives to conventional contact-based measurement methods for prefabricated box girders [15,16]. For example, Song et al. [17] constructed a 3D point cloud model of a 30 m highway concrete small box girder and applied surface-fitting analysis to identify section dimensions; however, their approach strongly depends on point cloud completeness and does not address the re-construction of missing regions. Wang et al. [18] developed a hybrid-pixel segmentation algorithm that, by optimizing edge-detection thresholds, substantially improves the geometric accuracy of boundary recognition for complex components, providing useful guidance for exterior-feature identification of large concrete elements. Zhao et al. [19] proposed a density-adaptive compression algorithm that effectively suppresses feature degradation in low-density regions and achieved an absolute error of 1.34 mm in the inspection of prefabricated concrete elements sized 400 mm × 400 mm × 600 mm, although its effectiveness for large-scale concrete components remains to be validated. Wu et al. [20] attempted to establish a full-process detection system for box girders but were unable to obtain complete 3D reconstruction because of missing point cloud data on the bottom and top faces. Wang et al. [21] exposed the limitations of uniform-grid compression algorithms in large-component inspection, showing that area-calculation errors grow nonlinearly with increasing component size. Ran, S.-C et al. [21] applied RFID technology to the appearance inspection of concrete structures, providing technical support and a theoretical basis for crack monitoring and detection.

A review of the existing literature indicates that current dimensional inspection studies based on 3D laser scanning are primarily focused on small- and medium-sized prefabricated components or local geometric feature analysis. However, several limitations remain in the inspection of large-scale high-speed railway precast box girders. First, multi-station point cloud datasets are extremely large, and conventional uniform downsampling methods tend to degrade critical edge features. Second, due to storage-yard occlusions and the structural characteristics of prestressed concrete girders, significant point cloud loss often occurs in key regions, while existing reconstruction methods struggle to balance geometric completeness with engineering authenticity. Third, the dimensional inspection process still relies heavily on manual intervention and lacks an automated inspection framework tailored to engineering acceptance requirements. Finally, comprehensive studies covering the entire inspection workflow for large-scale high-speed railway precast box girders remain limited, and a systematic technical framework has yet to be established.

To address these issues, this study takes the precast box girders of the Xiong’an–Xinzhou High-Speed Railway as the engineering background and proposes a multimodal point cloud optimization method for large-scale precast box girders. While preserving critical geometric features, the proposed method significantly improves the efficiency of large-scale point cloud processing. In addition, a point cloud reconstruction approach considering prestress-induced camber characteristics and geometric constraints in locally missing regions is developed, enabling high-accuracy restoration of incomplete point clouds in the bottom slab and flange regions of box girders. The proposed methodology provides theoretical support and technical assurance for the intelligent construction and prefabricated assembly of high-speed railway precast components.

## 2. Full-Process Inspection System for Large-Scale High-Speed Railway Prefabricated Box Girders

Accurate measurement of key parameters—such as span length and cross-sectional dimensions—during the acceptance stage of prefabricated high-speed-rail box girders constitutes the primary basis for product delivery. Conventional inspections, which rely on manual, contact-based instruments, suffer from low efficiency, numerous measurement blind spots, and incomplete data coverage, rendering them inadequate for the quality requirements of intelligent construction. Furthermore, storage areas within prefabrication yards are spatially constrained; the box girders themselves commonly exhibit an upward camber induced by prestressing, the top faces are densely occupied by embedded reinforcement, and the end regions feature protruding prestressing tendons and anchorage devices—all of which further complicate measurement. Therefore, the development of a fully automated, end-to-end inspection system—comprising system deployment, data acquisition, point cloud preprocessing, point cloud feature recognition and missing-region reconstruction, and dimensional identification—represents a critical breakthrough for improving box-girder quality control and expanding the applications of intelligent construction.

**Figure 1:**
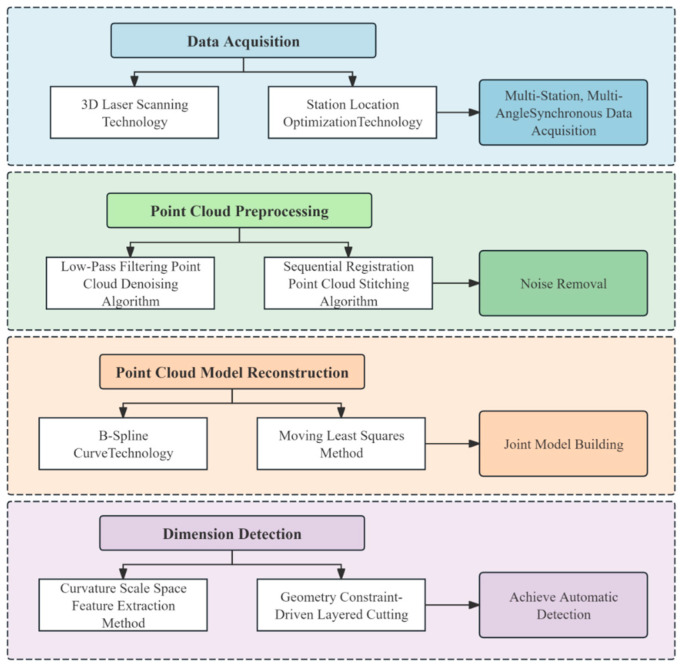
Overall workflow of the full-process inspection for prefabricated box girders.

To address the current limitations of point cloud detection technology—namely, the severe point cloud loss caused by occlusion from construction facilities and densely arranged double-layer beam storage in the data acquisition process; the insufficient adaptability of existing reconstruction algorithms for the beam’s non-fixed deflection surface characteristics; and the reliance on manual intervention in the dimensional extraction process, leading to fragmented workflows and poor structural repeatability—this study develops a full-process inspection system, as shown in Figure 1. First, a dynamic measurement station location optimization strategy for 3D laser scanning is established, which, combined with the spatial characteristics of the prefabricated beam yard and the geometric properties of the box girder, effectively avoids detection blind spots and significantly improves point cloud coverage. Second, a multimodal point cloud optimization method integrating low-pass filtering and sequential registration is developed to suppress noise from concrete surface textures while retaining key fine features, thereby improving data preprocessing’s efficiency and stability. Building on this, boundary points and curvature discontinuities are extracted based on the K-NN and PCA algorithms. For areas prone to missing data, such as the bottom slab and flange, B-spline curves and the MLS method are introduced for joint modeling, enabling deflection surface reconstruction that accounts for prestressing effects and symmetry repair. Furthermore, a hierarchical slicing method driven by geometric constraints, combined with CSS feature extraction, is employed to achieve the millimeter-level automatic detection of six critical dimensions, including beam length, deck width, and web thickness. This system significantly reduces manual involvement, increasing detection efficiency by approximately three times compared to manual methods. It effectively resolves the traditional issues of “experience-driven and inefficient” inspections, reducing the risk of beam misalignment caused by dimensional deviations.

Compared with existing studies, this work is the first to achieve a deep coupling between multi-scale geometric processing algorithms and the practical constraints of large-scale railway prefabricated box girders. It advances the inspection paradigm from passive spot-checking to active, full-feature analysis, and provides a more systematic, intelligent, and reusable technical pathway for the high-precision dimensional control of prefabricated components in high-speed rail projects.

## 3. Point Cloud Preprocessing Technology for Large-Scale High-Speed Railway Box Girders

During the 3D laser scanning of large-scale high-speed railway precast box girders, point cloud acquisition is subject to multiple constraints arising from both the spatial layout of the storage yard and the geometric characteristics of the girders themselves. Owing to the complex geometry, surface reflectivity, and on-site environmental conditions, the raw point cloud data often exhibit noise, non-uniform density, and local data voids. Therefore, preprocessing of the raw point cloud is essential prior to subsequent modeling and inspection to effectively suppress interference, recover structural features, and provide a reliable foundation for accurate point cloud reconstruction and key dimensional identification.

### 3.1. Low-Pass Filtering Point Cloud Denoising Algorithm

During the three-dimensional (3D) laser scanning of large prefabricated box girders, the acquired point cloud data are frequently contaminated by substantial noise. Such noise not only markedly increases the complexity of subsequent data processing and degrades overall data quality, but also impairs the accurate extraction of critical feature points, which in turn can cause reconstructed point cloud models to fail to meet the geometric-accuracy requirements of practical engineering applications. Therefore, the targeted denoising of point clouds for large box girders is of considerable practical significance.

Based on the spatial distribution characteristics of noise points, they can be categorized into four types: drift points, isolated points, redundant points, and mixed points. Drift points are located far from the main structure, sparsely distributed in space, and often suspended above the point cloud. Isolated points are anomalous points situated away from dense regions of the point cloud; they form small clusters and exhibit distributions that differ markedly from surrounding points. Redundant points mainly refer to extraneous points acquired outside the scanning range or duplicate records. Mixed points are distributed around the periphery of the target object; they are sparse and difficult to distinguish from genuine structural elements. For the first three types of noise, open-source software such as Cloud Compare 2.13.2 can be employed for screening and removal through multi-view visualization and manual interaction, for example, eliminating non-target point clouds corresponding to embedded reinforcement on box-girder sides or temporarily placed objects within the structure. However, because mixed points exhibit similar spatial distributions to those of target points and are therefore difficult to identify, manual processing is not only time-consuming and labor-intensive but also prone to the erroneous removal of valid point cloud data, which in turn degrades the completeness and accuracy of the reconstructed surface models.

**Figure 2:**
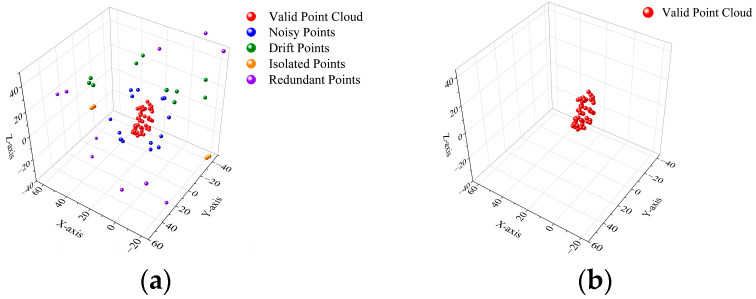
Denoising Comparison Diagram: (**a**) Before Point Cloud Denoising; (**b**) After Point Cloud Denoising.

To address this, we propose an automated denoising strategy grounded in the principle of low-pass filtering. The algorithm traverses the point cloud and, for each target point, selects either a preset number of nearest neighbors or the neighboring points within a specified radius *r*, and fits a local plane to that neighborhood. A maximum error threshold *w_r_* is introduced and the mean projection distance *d_m_* of the neighborhood points onto the fitted plane is computed; if *d_m_* exceeds *w_r_*, the target point is classified as noise and removed. As shown in Figure 2, the resulting comparison illustrates the point cloud denoising after removal of the various noise types. By using the neighborhood plane-projection error as the primary criterion, the method achieves the precise identification of noise points while avoiding the loss of valid points associated with manual cleaning, thereby preserving the geometric integrity of the box-girder main structure. Furthermore, the automated workflow increases denoising efficiency by a factor of 3–5 compared with manual interactive deletion, making it well suited to large-scale engineering point cloud processing.

### 3.2. Sequential Registration Point Cloud Stitching Algorithm

**Figure 3:**
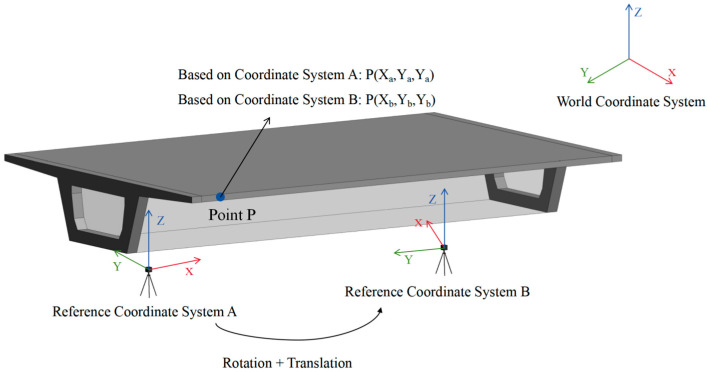
Point Cloud Stitching Principle.

Due to the geometric configuration and large spatial scale of prefabricated high-speed railway box girders, 3D laser scanning from a single station cannot fully capture all surfaces; therefore, multiple scanning stations must be deployed to acquire a complete, global point cloud. Data from each station are referenced to a local coordinate system centered on that scan, which leads to inconsistent coordinate frames across stations. To ensure spatial consistency of the assembled point cloud, a spatial–datum unification algorithm is applied to normalize the point cloud datasets from all stations [22]. Through inter-station coordinate transformation and registration, a complete point cloud model covering the entire box girder is constructed to satisfy the engineering requirements for subsequent inspection and analysis. The basic principle is illustrated in Figure 3.

As shown in Figure 3, the two scanning stations are defined in different local coordinate systems. Taking point P as an example, let its coordinates in station 1 and station 2 be (XA, YA, ZA) and (XB, YB, ZB), respectively. The coordinate transformation can then be expressed as

(1) $$R=\left[\begin{array}{ccc} \cos\alpha & -\sin\alpha & 0\\ \sin\alpha & \cos\alpha & 0\\ 0 & 0 & 1 \end{array}\right]\left[\begin{array}{ccc} \cos\beta & 0 & -\sin\beta \\ 0 & 1 & 0\\ \sin\beta & 0 & \cos\beta \end{array}\right]\left[\begin{array}{ccc} 1 & 0 & 0\\ 0 & \cos\gamma & -\sin\gamma \\ 0 & \sin\gamma & \cos\gamma \end{array}\right]$$

(2) $$T=\left[\begin{array}{c} t_{x}\\ t_{y}\\ t_{z} \end{array}\right]$$

In the above expression, *α*, *β*, *γ* denote rotation angles about the *x*, *y*, and *z*, respectively, while *t_x_, t_y_*, *t_z_* denote translations along the *x*, *y*, and *z* directions.

**Figure 4:**
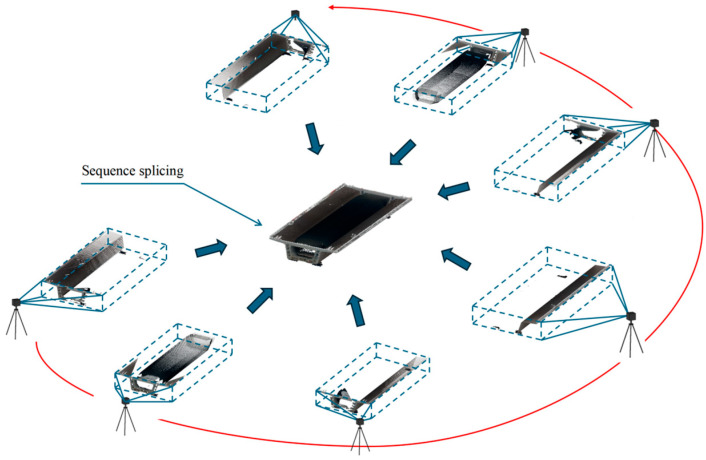
Point Cloud Stitching Diagram.

In this study, a sequential registration strategy was adopted to perform station-by-station merging of point clouds acquired from adjacent stations located at end faces, chamfers and similar positions. Given the high overlap between neighboring scans and the pronounced geometric features of the box-girder edges, automated registration implemented in commercial point cloud software (Cloud Compare) is appropriate: local feature descriptors are extracted from each station’s point cloud, initial transformation parameters are obtained via feature matching to achieve a coarse alignment, and a subsequent bundle-adjustment step is applied to globally optimize registration errors, thereby improving alignment accuracy and overall consistency. This approach obviates bulk import of all point cloud data and thus substantially reduces memory usage and computational load. Figure 4 presents the resultant integrated point cloud of the prefabricated concrete box girder obtained by stitching scans from different station angles.

### 3.3. Multimodal Point Cloud Simplification Technology

As a representative large-scale precast concrete element, prefabricated box girders may extend longitudinally for tens of meters, and their three-dimensional laser-scanned point clouds are typically acquired at millimeter-level sampling density, yielding extremely large raw datasets. The coexistence of high-curvature sharp edges, localized fine-scale features and extensive planar regions on the box-girder surface produces pronounced spatial non-uniformity: complex curved regions are densely sampled while planar areas are comparatively sparse. In addition, the scanning process frequently generates outliers and redundant points caused by repeated structural elements, further exacerbating data irregularity. The direct processing of such raw point clouds therefore encounters dual bottlenecks of limited storage capacity and poor computational efficiency. Consequently, achieving an efficient data-reduction scheme that preserves critical geometric features is a necessary prerequisite for subsequent modeling and dimensional inspection.

Numerous point cloud simplification methods have been developed, most of which are centered on geometric features such as inter-point distance, surface normals and curvature. In general, these methods can be classified into two main categories: mesh-based compression approaches and point cloud-based compression approaches [23]. Considering the characteristics of prefabricated box-girder point clouds in terms of scale, density and spatial distribution, and aiming to preserve geometric fidelity while maintaining computational efficiency, this study proposes a multimodal point cloud optimization technique for efficient box-girder point cloud reduction. The proposed technique integrates multi-scale voxel filtering with a curvature-sensitive enhancement strategy to perform down-sampling within a multi-dimensional data-processing framework. Specifically, a voxel-filtering method is first employed for coarse compression by partitioning the scanning space into regular cubic grids of a prescribed size and performing preliminary down-sampling within each grid. When the voxel edge length v is set to 5 mm, the three-dimensional space is divided into cubic cells with 5 mm edges, and all points within each cell are replaced by their centroid P, such that

(3) $$P=(\frac{1}{N}\sum_{i=1}^{n}x_{i},\frac{1}{N}\sum_{i=1}^{n}y_{i},\frac{1}{N}\sum_{i=1}^{n}z_{i})$$

In this expression, *n* denotes the number of points within a voxel, and $x_{i}$, $y_{i}$, and $z_{i}$ represent the three-dimensional coordinates of the i-th point inside the voxel.

The above approach markedly reduces data volume while preserving the overall geometric shape: a 5 mm voxel resolution retains macroscopic dimensional fidelity (e.g., web thickness) while alleviating redundancy in planar regions.

After the coarse compression effected by voxel filtering, a curvature-sensitive enhancement strategy is applied for fine-scale optimization. This strategy first computes local curvature using a covariance-matrix method, and defines a scalar C_λ_ to characterize the curvature variation at each point. The algorithm then iterates over the preliminarily sampled points p and evaluates the corresponding C_λ_ for each. For a given point p, the neighboring point set is searched within a radius r, and the covariance matrix *Cov* of that neighborhood is constructed as

(4) $$Cov=\frac{1}{k}\sum_{i=1}^{k}\left(p_{i}-P\right)(p_{i}-P{)}^{T}$$

In this expression, P denotes the centroid of the neighborhood points, and pi represents the coordinate vector of the i-th point within the neighborhood.

Eigenvalue decomposition is then performed on the covariance matrix Cov, yielding eigenvalues λ_1_ ≥ λ_2_ ≥ λ_3_ ≥ 0, based on which the curvature descriptor C_λ_ is subsequently computed as

(5) $$C_{\lambda }=\frac{\lambda_{3}}{\lambda_{1}+\lambda_{2}+\lambda_{3}}$$

**Figure 5:**
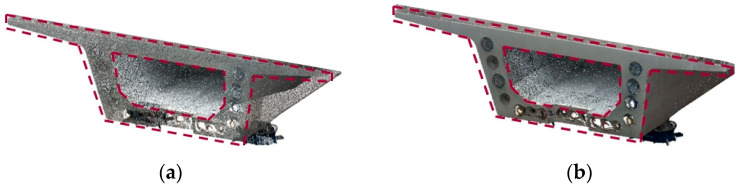
Point Cloud Simplification Effect Diagram: (**a**) Before Point Cloud Simplification; (**b**) After Point Cloud Simplification.

Finally, high-curvature points are selected according to a predefined curvature threshold to preserve feature-rich regions, thereby completing the point cloud simplification process. The resulting effect is illustrated in Figure 5.

## 4. Point Cloud Model Reconstruction Method for Large-Scale Box Girders

Owing to their large spans, thin walls and complex curved geometries, box girders generate laser-scanned point clouds that contain numerous edge-contour points, convex–concave transition surfaces and corner (curvature-discontinuity) points. These regions exhibit pronounced variations in surface normals that contrast sharply with smoothly curved areas and therefore provide a reliable basis for extracting salient geometric features and identifying key structural elements such as feature surfaces and feature lines. At the same time, the scanning environment in prefabrication yards is challenging: occlusion by supports, specular reflection from concrete surfaces, and the limited field of view and ranging capabilities of scanners commonly cause missing data in critical regions (for example, the bottom slab), which in turn induces geometric distortions in subsequent modeling such as surface discontinuities and blunting of sharp feature edges. Accordingly, there is an urgent need to develop reconstruction methods tailored to box-girder point cloud characteristics—advancing topology-aware connectivity, salient-feature detection and local data-completion techniques—to provide robust geometric models for subsequent high-precision dimensional inspection.

### 4.1. Box-Girder Point Cloud Feature-Recognition Algorithm

During box-girder point cloud processing, the raw data consist solely of discrete three-dimensional coordinates and color attributes, without explicit topological relationships among points. This absence of topology not only constrains the efficiency of geometric-feature extraction but also increases the computational complexity of subsequent tasks such as surface reconstruction. To address these issues, we propose a combined approach that integrates neighborhood-based topological modeling with multi-dimensional geometric-feature enhancement. By constructing neighborhood relationships within the point cloud, the method establishes an explicit topological structure and leverages local geometric descriptors to enhance salient features.

**Figure 6:**
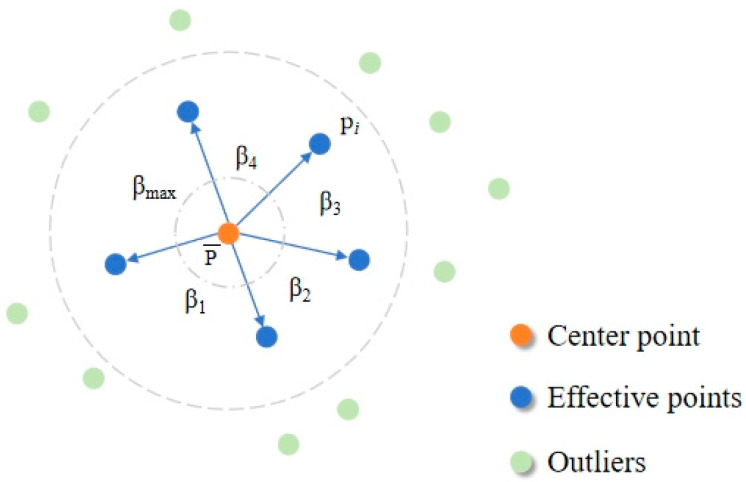
K-NN Relationship Model Diagram.

In this study, a K-NN algorithm is employed to determine whether a point Pi is a boundary point. The decision criterion is based on the angle between directed line segments formed by Pi and its neighboring points. This angle is compared with a predefined angular threshold: if the angle exceeds the threshold, Pi is classified as a boundary point; otherwise, it is regarded as an interior point. A schematic illustration of this principle is shown in Figure 6.

For boundary regions of the box-girder point cloud characterized by pronounced curvature discontinuities, PCA is further introduced to integrate normal-vector computation with curvature estimation, enabling accurate boundary identification. The core idea is to recast the estimation of the normal vector at a target point P as the problem of determining the normal of its local tangent plane. Specifically, a K-NN search is employed to select the neighborhood point set N = {n_i_|n_i_ ∈ N}, i = 1,2, …, k, where k denotes the number of neighboring points. The centroid of the neighborhood, $\bar{p}$, is then computed as

(6) $$\bar{p}=\frac{1}{k}\sum_{i=1}^{k}p_{i}$$

The normal vector of the least-squares fitted surface is computed according to the following expression:

(7) $$\begin{array}{c} \eta \\ min\sum_{i=1}^{k}∥(p_{i}-\bar{p})\vec{n} \end{array}$$

where $\vec{n}$—the normal vector of the fitted plane.

The minimization above is equivalent to solving an eigenvalue problem for the covariance matrix Cov; specifically, the normal vector at the target point is given by the eigenvector associated with the smallest eigenvalue of Cov:

(8) $$Cov=\sum_{i=1}^{k}\left(p_{i}-\bar{p}\right)(p_{i}-\bar{p}{)}^{T}$$

Eigenvalue decomposition is performed on the covariance matrix, yielding eigenvalues λ_1_, λ_2_ and λ_3_ together with their corresponding eigenvectors v_1_, v_2_, and v_3_.

The normals obtained from PCA are sign-ambiguous: if v is a principal direction then −v is equally valid, since both represent the direction of maximum variance. However, eigenvectors of the covariance matrix *Cov* carry no inherent positive or negative orientation and therefore must be reoriented with respect to the sensor (viewpoint) direction. This reorientation can be expressed as

(9) $$\vec{n}=\left\{\begin{array}{cc} n_{i} & n_{i}\cdot \left(v_{p}-q\right)>0\\ -n_{i} & else \end{array}\right\}$$

In this expression, v_p_ denotes the viewpoint, and q represents the sampled point.

The curvature *ϕ* is then computed as the ratio of the smallest eigenvalue to the sum of all eigenvalues, expressed as

(10) $$\phi =\frac{\lambda_{3}}{\lambda_{1}+\lambda_{2}+\lambda_{3}}$$

**Figure 7:**
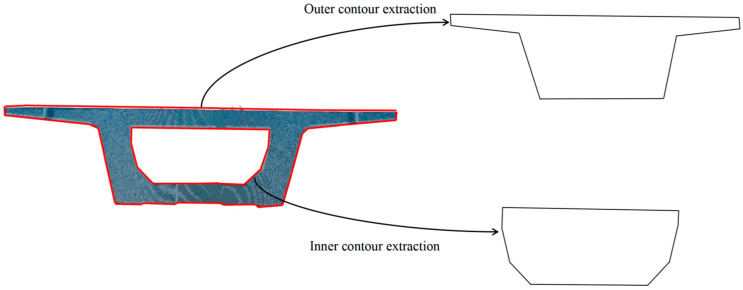
Box-Girder End-Face Boundary Extraction.

The above multi-scale approach effectively captures edge details across scales and consequently improves boundary accuracy; however, it also introduces additional iterations and redundant storage, which increase computational time and resource consumption. To improve efficiency, a detection-window parameter is provisionally set to 10 and the point cloud is traversed: points whose curvature values exceed the prescribed threshold are tentatively classified as boundary points. In the subsequent, coarser-scale pass a plane-fitting-based curvature estimator is used, and points previously classified as non-boundary are excluded from further computation. The boundary detection results for the box-girder end face are shown in Figure 7.

### 4.2. Filling of Missing Point Clouds for Box Girders

**Figure 8:**
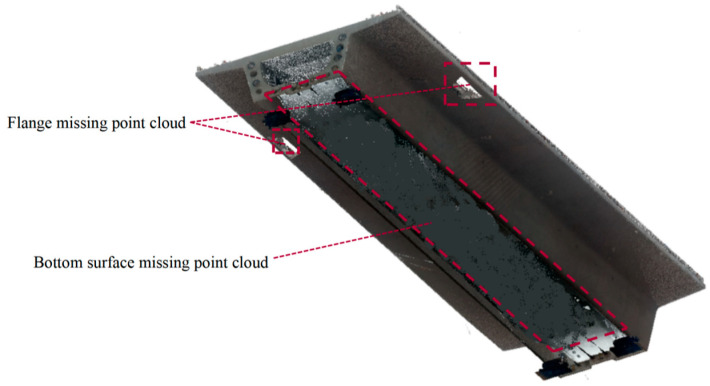
Wing Flange Side Missing Point Cloud.

During field acquisition of point clouds in the storage area of prefabrication yards, data incompleteness is particularly pronounced, primarily due to the combined constraints of scanning geometry and instrument performance. On the one hand, large on-site metal fixtures such as supports and beam-storage racks induce self-occlusion, and—when coupled with the high specularity of concrete surfaces—these effects exacerbate scanning blind spots. On the other hand, the limited field of view and maximum ranging capability of laser scanners impede complete capture of the full-surface geometry of wide-span elements such as the bottom slab, resulting in widespread missing data. Representative local patterns of such data loss are shown in Figure 8.

This study employs B-spline curve fitting, least-squares optimization and the iterative closest point (ICP) algorithm to fill and repair the box-girder soffit (underside) and local voids in incomplete point clouds. The proposed reconstruction restores the complete geometry of the box-girder point cloud, thereby facilitating the extraction of refined geometric measurements and providing a reliable geometric model for subsequent dimensional inspection.

#### 4.2.1. Filling of Box-Girder Bottom Surface

In precast box-girder construction, prestressing (tensioning) is employed to improve crack resistance and stiffness, but the induced forces can produce reverse-camber deformation so that the deck and soffit exhibit slight upward camber. Consequently, filling missing point cloud regions must reproduce the true deformed surface rather than substituting simple planar patches. Moreover, chamfered transitions are commonly provided at the junction between soffit and web to mitigate stress concentrations, and the reconstruction must preserve smooth geometric transitions in these areas. To meet these requirements, the soffit-repair procedure is as follows. First, the covariance matrix of the point cloud at the soffit–web interface is computed and high-curvature feature points are extracted using a curvature threshold of 0.1; the chamfer region is then segmented by clustering high-curvature points with an additional constraint on normal-vector angular difference, and a B-spline curve is fitted to obtain the chamfer boundary contour. Second, a chamfer surface is generated along the intersection line using the design chamfer radius R = 50 mm, and the overall soffit is reconstructed by a moving least-squares (MLS) surface-fitting step to ensure a smooth junction between the chamfer and the main soffit surface. Finally, during the connection-stage refinement, the generated chamfer surface is fused with the retained original point cloud and MLS-based local smoothing is applied to the stitching boundary; sampling along the chamfer edge is densified at the design radius to improve the localization accuracy of geometric features.

**Figure 9:**
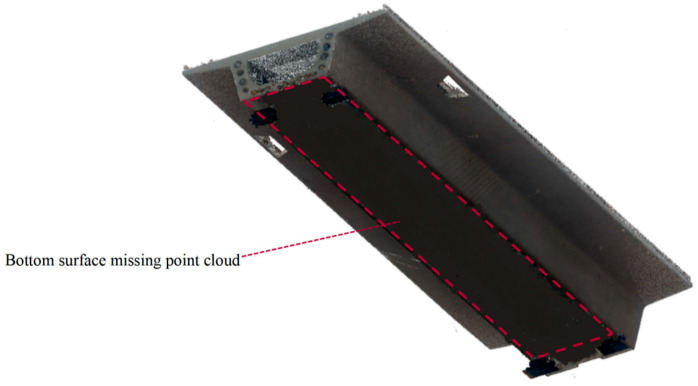
Box-Girder Bottom Surface Filling.

Following the above procedures, the reconstructed soffit surface closely conforms to the actual deformation of the box girder induced by prestressing while effectively mitigating stress-concentration risks at the chamfered transitions. The repair quality can be directly validated by three-dimensional point cloud visualization; the filled underside of the girder is shown in Figure 9.

#### 4.2.2. Wing Flange Void Repair

**Figure 10:**
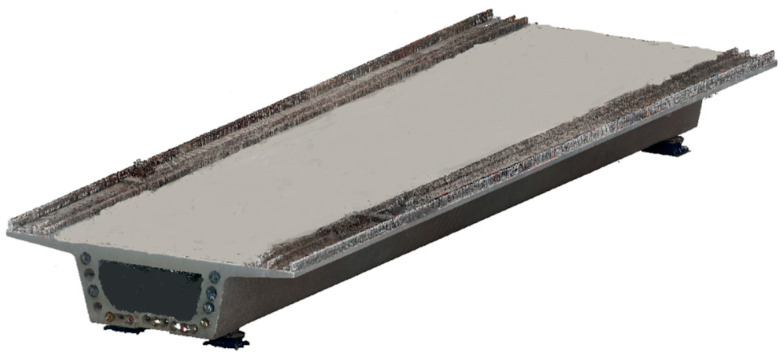
Complete Box-Girder Point Cloud Model.

To address point cloud data loss caused by embedded reinforcement in railway box-girder flanges, this study proposes a repair strategy that integrates geometric symmetry with surface re-construction, with particular emphasis on restoring single-sided missing regions formed by occlusion on concrete surfaces. First, PCA is used to extract the longitudinal principal direction of the box girder: the covariance matrix of the point cloud is computed, and the eigenvector corresponding to the largest eigenvalue is taken as the normal vector of the symmetry plane, enabling high-precision localization of the flange mid-symmetry plane. Next, this symmetry plane is used to perform a rigid transformation of the point cloud on the intact flange side—specifically, a mirror-mapping operation about the symmetry plane is applied to generate an initial filled point cloud for the missing side. This process, implemented via coordinate-system rotation and translation, strictly preserves geometric symmetry correspondence. To address potential geometric discontinuities between the mirrored surface and the original surface, feature control points P are introduced in the boundary region, and a cubic B-spline surface is employed to construct a transition zone. Owing to the local control properties of B-spline surfaces, smooth geometric transitions are achieved, avoiding abrupt changes between the repaired region and the original structure. Finally, to further improve reconstruction accuracy, an iterative closest point (ICP) algorithm is applied to optimize feature matching between the original and mirrored boundaries. Using point-to-surface distance as the metric, the rotation and translation parameters of the rigid transformation matrix T are iteratively refined until geometric consistency between the transition surface and the original structure is satisfied. By imposing symmetry constraints, the proposed method significantly reduces computational complexity, and in combination with B-spline surface optimization and ICP-based fine adjustment, effectively enhances the geometric accuracy and continuity of the repair results. This approach successfully resolves flange point cloud loss caused by occlusion from embedded reinforcement. The reconstructed overall point cloud model of the box girder is shown in Figure 10.

## 5. Feature-Driven Dimensional Inspection Method for Box Girders

To mitigate the high computational complexity and low feature-extraction efficiency associated with processing massive point cloud datasets of large-scale beams, practitioners commonly adopt a geometry-constrained, hierarchical slicing strategy to streamline the workflow [24,25]. In this study, and following the key measurement locations prescribed by beam-dimension inspection standards, cutting planes are placed along the beam’s longitudinal axis at fixed intervals (end section, L/5, 2L/5, 3L/5, 4L/5), partitioning the raw point cloud into multiple geometrically coherent subregions. Each subregion corresponds to the local structure of a specific beam segment; within these segments, the point-density distribution is more uniform and noise interference is effectively suppressed, thereby providing a more stable data foundation for subsequent feature extraction and dimensional computation.

**Figure 11:**
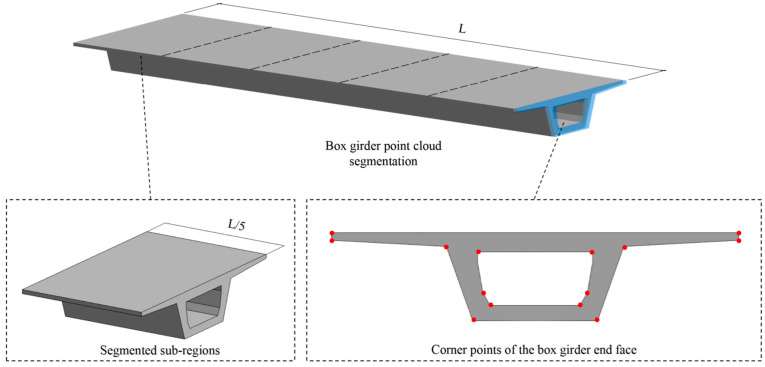
Point cloud segmentation and corner-point extraction workflow.

By orthogonally projecting the point cloud data on both sides of each cutting plane onto a reference plane, the three-dimensional point set is reduced to a two-dimensional representation, substantially lowering data volume while preserving key geometric features. This hierarchical slicing strategy not only aligns with the beam’s structural–mechanical characteristics—particularly the stress distribution at web–flange junctions—but also mitigates numerical singularities in global computations, thereby providing a more reliable data foundation for subsequent feature-line extraction and deformation analysis. A schematic of the box-girder point cloud segmentation model is shown in Figure 11.

### 5.1. Box-Girder Feature Corner Points

Prefabricated high-speed-rail box girders are characterized by hollow cross-sections and sharply defined edges. From the viewpoint of 3D point cloud feature analysis, the corner points at the beam ends exhibit several engineering-relevant properties. First, they show strong geometric correlation: these points concentrate along the intersection lines of primary load-bearing elements (web–flange and crossbeam–web junctions), and their spatial coordinates directly reflect the geometric convergence of the cross-section. Second, they possess excellent topological stability: during reverse-camber deformation induced by prestressing, displacements of the corner points display a pronounced linear relationship with overall profile deviations, making them sensitive indicators for deformation monitoring. Third, they offer superior detection robustness: compared with conventional total-station surveys that are prone to occlusion, corner points—being local extrema of geometric curvature—can be located with high precision by exploiting point cloud density and local geometric cues.

### 5.2. Box-Girder Feature Corner Point Recognition Method

After sectioning the beam point cloud, we further reduce data volume and precisely extract geometric features by projecting each slice onto a reference plane to obtain two-dimensional contour point sets. This dimensionality-reduction step substantially lowers data complexity while efficiently capturing component boundary features, thereby laying the foundation for the accurate corner-point extraction required for subsequent dimensional inspection. Informed by the end-geometry of the box girder, the feature corner points to be extracted are classified into two types: (1) intersections of two or more edge lines, and (2) junctions where three planes or surfaces meet. Together, these two classes constitute the geometric landmarks of the component’s critical locations.

Given the general applicability of the CSS corner detector to contour analysis—which, via edge detection or multi-scale curvature computation, is robust to rotation, translation and noise and can stably identify curvature extrema on contours of arbitrary shape—we adopt this algorithm for end-face corner extraction. In practice, the CSS algorithm is applied to the previously extracted edge-line data of the beam end; multi-scale curvature analysis is then used to select curvature extrema that satisfy the prescribed constraints, thereby extracting the key corner points that represent the end geometry of the box girder. The computational procedure is as follows: first, acquire the boundary point cloud of the box-girder end and reconstruct the edge line, converting the boundary into a continuous curve parameterized by arc length s, (x(s), y(s)); second, smooth the curve on the reference plane by applying Gaussian filters at multiple scales X(s,σ)=x(s)⋅G(s,σ), chosen to cover both the largest and smallest corner features, and separately filter the x- and y-components to obtain X(s,σ):

(11) X(s,σ)=x(s)·G(s,σ)

(12) Y(s,σ)=y(s)⋅G(s,σ)

For a given parametric vector representation of a planar curve, the curvature is computed as follows:

(13) $$k(s)=\frac{\dot{x}(s)\ddot{y}(s)-\ddot{x}(s)\dot{y}(s)}{({\dot{x}}^{2}(s)+{\dot{y}}^{2}(s){)}^{\frac{3}{2}}}$$

In the expression, $\dot{x}$ and $\dot{y}$ denote first derivatives, $\ddot{x}$, and $\ddot{y}$ denote second derivatives, and s denotes the arc length between two points along the curve.

(14) $$s=\int_{P_{1}}^{P_{2}}\sqrt{{\dot{x}}^{2}(s)+{\dot{y}}^{2}(s)}dt$$

When the curve is parameterized by s, that is, ${\dot{x}}^{2}(s)+{\dot{y}}^{2}(s)=1$, the curvature in the CSS framework for an arc-length parameterized planar curve can be expressed as

(15) $$k(s)=\dot{X}(s)\ddot{Y}(s)-\ddot{X}(s)\dot{Y}(s)$$

For curvature maxima across multiple scales, curvature extrema with small absolute values are first identified and discarded. The remaining curvature maxima are then screened: a point that is detected in adjacent scales is classified as a stable feature. Spatially proximate detections are merged via clustering. By referencing the end-geometry and design drawings of the box girder, corner points that do not correspond to design-intended locations are removed; the retained corner points are overlaid on the original imagery and their validity is confirmed through interactive human–computer verification. The corner-point detection results for the box-girder end face are shown in Figure 11.

## 6. Rationality Verification and Engineering Application of Large-Scale Prefabricated Box-Girder Inspection Methods

### 6.1. Project Overview

**Figure 12:**
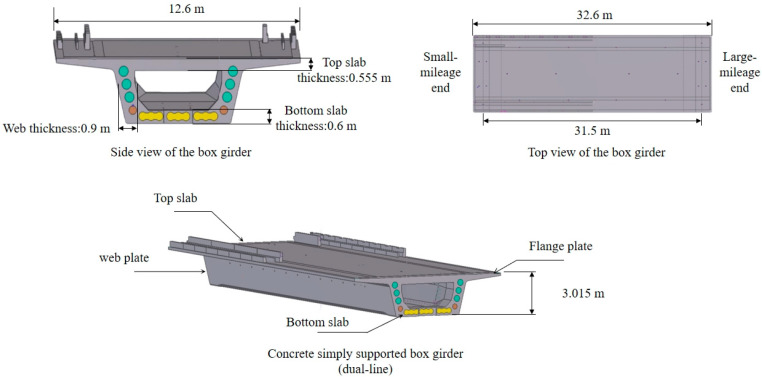
Box-Girder Inspection Information.

This study investigates 31.5 m post-tensioned prefabricated box girders used in the Xiong’an–Xinzhou high-speed railway. In storage yards, these girders experience dynamic changes in alignment due to prestressing forces and concrete creep, making direct modeling based on design drawings impractical and necessitating in situ geometric dimension inspection. Measurements were conducted at the Wutai beam fabrication yard of the Xiong’an–Xinzhou high-speed railway; the inspected girders are shown in Figure 12. A total of nine prefabricated girders were randomly selected for appearance-dimension inspection, labeled WT-01 to WT-09. According to Prefabricated Post-tensioned Prestressed Concrete Simply Supported Beams for High-speed Railways (GB/T 37439-2019) [26] and related bridge and culvert acceptance standards, finished-beam acceptance requires measurements of overall beam length, deck width, web thickness, beam depth, and top- and bottom-slab thicknesses. The design values and allowable tolerances for each inspection item are listed in Table 1.

### 6.2. Scanner Selection and Station Layout

#### 6.2.1. Equipment Selection

In large-scale prefabricated box-girder construction, member lengths commonly reach 24 m, 32 m and even up to 40 m, imposing stringent requirements for high-precision, high-efficiency 3D data acquisition in storage yards. In practice, however, the completeness, accuracy and reliability of point cloud data are substantially challenged by factors such as scanner range, incidence angle, scanner-station layout and prevailing climatic conditions. Consequently, equipment selection must strike a balance among scanning coverage, compatibility with target dimensions and project budget to ensure both the technical feasibility and cost-effectiveness of the chosen solution.

After a systematic evaluation, this study selected the portable SPL-1500 3D laser scanner for point cloud data acquisition. This device offers efficient scanning capabilities for medium and long-range distances, with a scanning range of 1.5 m to 1500 m and a measurement speed of up to 2 million points per second. It features a highly integrated hardware design, with an overall weight of only 4.85 kg and built-in dual-axis compensation, enabling quick operation without the need for leveling. Additionally, the scanner is equipped with dual 12.3-megapixel high-resolution cameras for fast color rendering of the real scene. It also integrates multiple sensors, including GNSS, an electronic compass, an altimeter, and a thermometer, for real-time monitoring of the device’s status. The scanner provides multimodal hardware interfaces and standardized communication protocols, allowing for easy integration with various application systems, offering excellent compatibility and effectively adapting to the complex construction environment of beam yards.

#### 6.2.2. Station Layout

Before conducting 3D laser scanning in the storage area for prefabricated box girders, a comprehensive survey of the storage yard environment must first be conducted. The spatial location of the scanning stations and the distance from the stations to the target beams should be scientifically planned, taking into account the geometry and dimensional parameters of the prefabricated beams, as well as the technical specifications of the scanner, including range and measurement accuracy. This ensures that the station layout fully covers key feature areas on both the end faces and sides of the beams while effectively avoiding scanning blind spots or redundancy caused by excessive overlap of point cloud data.

Traditional 3D scanning station deployment schemes typically rely on strategically placing common targets, which serve as a coordinate transfer reference between multiple stations. Through precise point cloud registration and stitching, a complete 3D model is generated. These methods can generally be categorized into two types: tail-to-tail registration for two stations and global registration for multiple stations [27]. With the ongoing iteration of point cloud registration algorithms, continuous improvements in hardware performance, and the increasing demand for efficiency and automation in complex engineering environments, 3D scanning technology has evolved to include a target-free scanning mode. This method eliminates the need for physical targets and instead uses algorithms to match 3D data features in overlapping areas between multiple stations, aligning the spatial coordinates directly. For prefabricated box girders, which have prominent edges and regular geometric features, the data-processing process requires only the extraction and matching of corresponding feature points between different stations to complete the stitching. Compared with traditional target-based methods, target-free scanning eliminates the need for control points or targets, significantly improving field inspection efficiency. However, this method has stricter requirements for the extent of overlapping areas between scanning stations, with a recommended overlap of 20–30% to ensure the accuracy of registration meets engineering needs.

**Figure 13:**
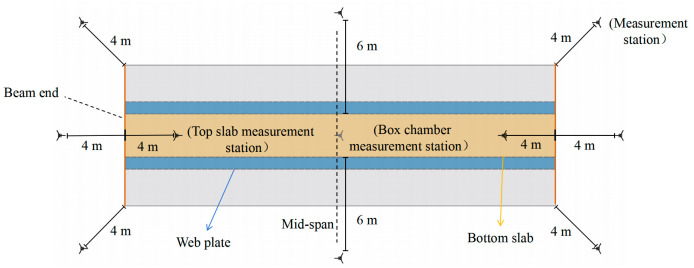
Scanning Station Layout.

To balance the efficiency and completeness of 3D laser scanning data acquisition, and to address the structural characteristics of the box girder as well as the need for compensating for potential scanning blind spots, this study adopts a multi-view collaborative stationing strategy, setting up 11 stations in total. The specific station layout is shown in Figure 13. The station locations and their respective functions are as follows: (1) End Region: Two stations are symmetrically placed approximately 4 m from the beam ends to ensure complete capture of the end geometry and avoid blind spots caused by curvature discontinuities; (2) Side Region: A station is placed at the mid-span cross-section of the beam, offset about 6 m outward along the web centerline, effectively covering the complex curves at the web–flange junction and minimizing local occlusions from changes in web thickness; (3) Top Slab Region: A station is located on the longitudinal symmetry axis of the top slab, 4 m from each end, using a downward scanning mode to capture high-resolution data of the slab’s flatness and reinforcement features; (4) Interior of the Box Girder: An additional station is set at the mid-span cross-section to focus on capturing detailed features inside the box, such as the internal chamfer and diaphragms.

The point cloud overlap between adjacent stations is maintained above 20% to ensure seamless stitching after data registration. For blind spots formed by the obstruction of the soffit due to supporting structures, surface fitting is applied to the overlapping areas of adjacent stations. This is combined with geometric constraints, such as the web edge lines and flange profiles, and optimized using the ICP algorithm to achieve high-precision data reconstruction for the missing regions.

### 6.3. Point Cloud Data Acquisition and Analysis

After completing the multi-view collaborative stationing plan and setting up the stations, 3D laser scanning of the beam is carried out. Since the raw point cloud data from a single station typically exceeds 1.5 million points, the first step to enhance processing efficiency and accuracy is to use the open-source software Cloud Compare for interactive denoising. This process removes redundant point clouds outside the target beam’s range and large noise clusters caused by environmental interference. Next, a low-pass filter is applied to eliminate outlier noise points, reducing the point cloud data volume for a single beam. For segmented beam point clouds collected from multiple stations, a semi-automated stitching process is implemented based on a sequential registration algorithm, ultimately integrating the data into a complete point cloud model that covers the entire beam.

Given the issue of excessive data volume in the stitched complete point cloud, coarse and fine compression processes are sequentially applied. Building on the contour extraction and feature calculation methods introduced in Section 3 and Section 4, operations such as beam profile boundary extraction, missing point cloud completion, point cloud segmentation, and feature point extraction are performed in succession, enabling precise calculation of the various dimensional inspection parameters.

#### 6.3.1. Validation Experiment

To validate the practical effectiveness of the proposed station-layout optimization scheme and point cloud processing algorithms for dimensional inspection of 32 m precast box girders, a comparative experiment was conducted. Two measurement methods, namely 3D laser scanning and conventional manual measurement, were employed to inspect six key acceptance indicators of nine precast box girders, including total girder length, deck width, web thickness, girder height, top slab thickness, and bottom slab thickness.

**Figure 14:**
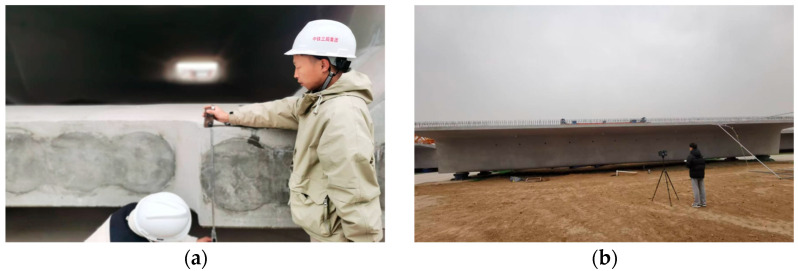
Comparison of Detection Processes: (**a**) Manual Measurement Monitoring; (**b**) 3D Laser Scanning Monitoring.

**Figure 15:**
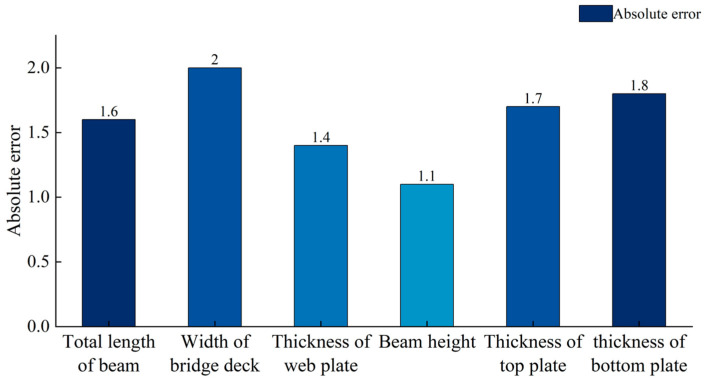
Average absolute errors of dimensional measurements.

The conventional manual measurement method utilized standard surveying instruments such as levels and steel tapes, with each girder measured individually according to established procedures. The corresponding inspection workflow is illustrated in Figure 14. In contrast, the 3D laser scanning method generated a high-precision full-girder model based on the previously described multi-view station arrangement and point cloud processing workflow, from which the required dimensions were automatically extracted. The average absolute errors and standard deviations between the two methods are presented in Table 2, and the comparison results are shown in Figure 15.

As shown in Figure 15, after point cloud denoising, registration, compression, and feature extraction, the geometric fidelity of dimensional information was effectively preserved. The results demonstrate that the proposed point cloud-based dimensional inspection method provides high measurement accuracy and reliability, with all average absolute errors remaining within 2.0 mm.

To further evaluate the engineering efficiency of the proposed method, the time consumption of each stage in the laser scanning workflow was statistically analyzed and compared with that of conventional manual inspection. The results indicate that the 3D laser scanning method significantly reduces inspection time. During field data acquisition, 11 scanning stations were arranged, with approximately 3 min required for each station, resulting in a total acquisition time of 33 min. Subsequent point cloud processing and dimensional analysis required approximately 60 min. Therefore, the complete inspection and analysis process for a box girder could be completed within approximately 1.5 h.

In contrast, the efficiency of manual inspection is strongly influenced by operator experience, team coordination, and measurement procedures, typically requiring more than 4 h to complete. Detailed comparisons of inspection efficiency are summarized in Table 3. The proposed method improves processing efficiency by approximately 3–5 times, significantly enhancing the acceptance inspection efficiency of precast box girders.

#### 6.3.2. Beam Exterior Dimensional Accuracy Testing

**Figure 16:**
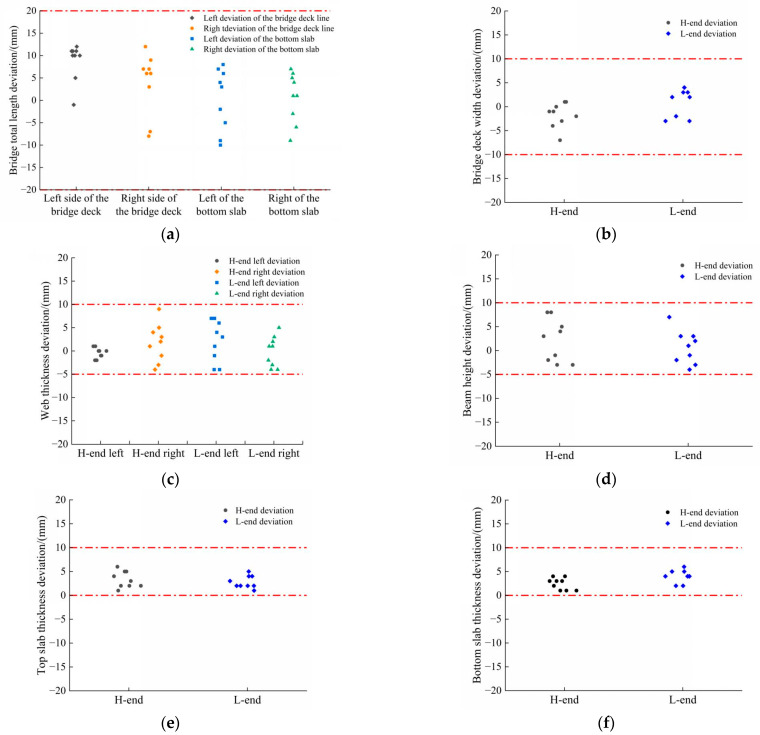
Detection Deviation: (**a**) Beam Number–Beam Length Deviation; (**b**) Beam Number–Deck Width Deviation; (**c**) Beam Number–Web Thickness Deviation; (**d**) Beam Number–Beam Height Deviation; (**e**) Beam Number–Top Slab Thickness Deviation; (**f**) Beam Number–Bottom Slab Thickness.

After processing the point cloud data of the nine girders, the beam number–deviation scatter plot shown in Figure 16 was generated. As can be seen from the Figure, the measurement deviations for certain exterior dimensions of the box girder—such as overall beam length, deck width, web thickness, beam height, top-slab thickness, and bottom-slab thickness—obtained using the 3D laser scanning technology all fall within the allowable tolerance limits specified in Table 1. This demonstrates that the method meets the predefined accuracy and reliability requirements for practical engineering inspections.

### 6.4. Measurement Uncertainty and Applicability Analysis

#### 6.4.1. Measurement Uncertainty Analysis

To further evaluate the reliability of the proposed 3D laser scanning-based dimensional inspection method for precast box girders, a measurement uncertainty analysis framework was established to quantify the major sources of error throughout the entire process of point cloud acquisition, processing, and dimensional extraction. Unlike conventional manual measurements, the proposed inspection system involves multiple processing stages, including point cloud acquisition, registration, filtering, reconstruction, and feature extraction, each of which may contribute to the final measurement uncertainty. Therefore, it is necessary to decompose and quantify the contribution of individual error sources.

Based on the inspection workflow adopted in this study, the primary sources of measurement uncertainty include scanner ranging error, multi-station point cloud registration error, point cloud filtering error, missing-region reconstruction error, and feature extraction error. Scanner error is mainly determined by the intrinsic ranging accuracy of the laser scanning device. Registration error originates from spatial transformation deviations during feature matching within overlapping regions between adjacent scanning stations. Filtering error is caused by partial geometric information loss during noise removal and point cloud compression. Reconstruction error arises from geometric approximation during surface fitting and the completion of missing regions. Feature extraction error is associated with discretization effects in corner-point detection and dimensional calculation.

According to the law of uncertainty propagation, the combined standard uncertainty can be calculated using the root–sum–square (RSS) method:

$$u_{c}=\sqrt{u_{1}^{2}+u_{2}^{2}{+u}_{3}^{2}{+u}_{4}^{2}{+u}_{5}^{2}}$$

where (c) denotes the combined standard uncertainty, and $u_{1}\sim u_{5}$ represent the standard uncertainties associated with scanner ranging, point cloud registration, filtering, reconstruction, and feature extraction, respectively.

Based on statistical analysis of the experimental data, the contributions of individual uncertainty sources are summarized in Table 4.

As shown in Table 4, point cloud reconstruction error and scanner ranging error are the dominant contributors to the overall uncertainty, accounting for more than 70% of the total uncertainty. Reconstruction error is mainly concentrated in locally missing regions such as flange and bottom slab areas, whereas registration error is primarily influenced by station overlap ratio and point cloud quality.

Using a coverage factor of (k = 2), the expanded uncertainty can be calculated as

$$U=k\cdot u_{c}$$

The expanded uncertainty of the proposed inspection system is therefore approximately ±3.2 mm. Furthermore, analysis of the dimensional inspection results from nine precast box girders showed that all measurement errors were controlled within 2.0 mm, demonstrating the high stability and reliability of the proposed point cloud-based inspection framework in practical engineering applications.

#### 6.4.2. Applicability and Limitations of the Proposed Method

The proposed 3D laser scanning-based dimensional inspection method is primarily applicable to the dimensional assessment of regular and symmetric structures, such as prestressed concrete simply supported box girders. Key dimensional parameters, including girder length, deck width, web thickness, girder height, top slab thickness, and bottom slab thickness, can be automatically identified and measured with millimeter-level accuracy.

From the perspective of point cloud quality, the proposed method is most suitable for engineering scenarios with relatively low levels of point cloud incompleteness. When the missing area accounts for less than 15% of the total surface area, satisfactory reconstruction results can be achieved through B-spline surface fitting and geometry-constrained completion. However, when the missing area exceeds 30%, the uncertainty of the reconstructed model increases significantly, potentially affecting the accuracy of subsequent dimensional measurements.

In addition, the proposed flange reconstruction strategy is based on the global symmetry characteristics of box-girder structures and demonstrates excellent applicability to standard prestressed concrete simply supported box girders. However, for special structures exhibiting significant local deformation, large-scale non-uniform creep deformation, or structural damage, the symmetry assumption may no longer be valid. In such cases, additional control-point constraints or deformation compensation models may be required to improve reconstruction accuracy.

It should be noted that the validation conducted in this study was performed in a precast girder production yard, where the scanned objects were concrete box girders with regular geometry and continuous surfaces. For steel bridges, highly reflective structural components, and complex irregular geometries, the point cloud acquisition characteristics and reconstruction mechanisms differ considerably. Therefore, the applicability of the proposed method to such structures requires further investigation.

Overall, for large-scale box girders with stable global geometric characteristics, the proposed method enables efficient and reliable dimensional inspection and exhibits strong potential for practical engineering applications.

## 7. Conclusions

This study addresses long-standing issues in the inspection of large-scale railway prefabricated box girders, such as reliance on manual contact measurements, low levels of automation, and inadequate result visualization. Leveraging the engineering practice at the Wutai beam fabrication yard of the Xiong’an–Xinzhou high-speed railway, an innovative, full-process non-contact inspection system was developed. This system covers data acquisition, processing, reconstruction, and dimensional inspection.

(1) In the data preprocessing stage, low-pass filtering, sequential registration, and multimodal point cloud optimization techniques were employed to process the box-girder point cloud data. Combined with a curvature-adaptive downsampling strategy, the proposed approach improved data processing efficiency by approximately 3–5 times compared with the original point cloud processing workflow under the data scale considered in this study.
(2) To address the challenges of complex structural geometry, scanning occlusions, and incomplete data in the reconstruction of large-scale box-girder point cloud models, a point cloud reconstruction method integrating topological relationship modeling and multi-scale feature enhancement was proposed. Through multi-scale feature enhancement, smooth surface transition, and data completion strategies, the geometric continuity of missing regions was significantly improved, providing a more complete point cloud model for subsequent dimensional inspection.
(3) A hierarchical slicing strategy guided by geometric constraints for large-scale railway prefabricated box girders is developed. The CSS algorithm is used to automatically identify eight key geometric features, and projection-based dimensionality reduction is employed to efficiently extract feature corner points.
(4) Comparative measurements were conducted between the proposed laser scanning method and the conventional manual measurement approach in an actual engineering project. The results indicate that the proposed method can achieve millimeter-level dimensional inspection accuracy under the experimental conditions of this study. Validation on nine 32 m precast box girders showed that the absolute errors between the measured dimensions and the corresponding manual measurements were all less than 2.0 mm, satisfying the accuracy requirements for dimensional inspection of large-scale precast box-girder structures.

The results of this study were successfully applied to the finished-product inspection of prefabricated box girders at the Wutai beam fabrication yard of the Xiong’an–Xinzhou high-speed railway. This facilitated the practical application of non-contact data acquisition technologies such as 3D laser scanning and automated analysis algorithms in the quality inspection of bridge engineering prefabricated components, demonstrating strong engineering adaptability and significant potential for widespread implementation.

## Figures and Tables

**Table 1:** Design values and allowable tolerances for inspection items.

No.	Item	Design Value (mm)	Allowable Tolerance (mm)
1	Span	31,500	±20
2	Overall beam length	32,600	±20
3	Deck width	12,600	+10, −10
4	Web thickness	900	+10, −5
5	Beam depth	3015	+10, −5
6	Top slab thickness	555	+10, −0
7	Bottom slab thickness	600	+10, −0

**Table 2:** Comparison of dimensional inspection results between 3D laser scanning and manual measurement.

Item	Average Absolute Error (mm)	Standard Deviation (mm)
Total girder length	1.6	0.7
Deck width	2.0	0.8
Web thickness	1.4	0.6
Girder height	1.1	0.5
Top slab thickness	1.7	0.8
Bottom slab thickness	1.8	0.9

**Table 3:** Efficiency comparison between manual inspection and 3D laser scanning inspection.

Item	Manual Inspection	Laser Scanning Inspection
Total girder length	30 min	6 min
Deck width	25 min	6 min
Web thickness	30 min	3 min
Girder height	25 min	6 min
Top slab thickness	20 min	9 min
Bottom slab thickness	20 min	3 min
Data processing	90 min	60 min
Total working time	4 h	1.5 h

**Table 4:** Major uncertainty sources and corresponding standard uncertainties.

Error Source	Standard Uncertainty (mm)
Scanner ranging error	0.8
Point cloud registration error	0.6
Point cloud filtering error	0.3
Point cloud reconstruction error	1.1
Feature extraction error	0.5
Combined standard uncertainty	1.6

## Data Availability

The authors declare that they have no known competing financial interests or personal relationships that could have appeared to influence the work reported in this paper.
